# Book Reviews

**Published:** 1896-09

**Authors:** 


### BOOK REVIEWS.

---

Merck's 1896 Index. "Encyclopedia for the Physician and Pharmacist." Second edition, pp. 268. $3.00.

This "index" contains all necessary information concerning chemicals and drugs used in medicine, in chemistry, and in the arts. It is a useful book, especially for those desirous of obtaining information upon the composition and actions of the newer drugs.

---

Grande Librairie Médicale A. Maloine. Place et Rue de l'École-de-Médecine, 21-23-25. Paris. Des Angines Couenneuses non Diphtériques. Considération sur la Pathogénie, le Diagnostic et le Traitement par le Docteur Dufaud, Médecin-Major de 2e Classe. 1 Volume in-18. 1 Fr. 50.

This little volume of rather more than 100 pages, including an account of twenty-eight cases and a bibliography, is written with the intention of assisting practitioners in the diagnosis of diphtheria and the diseases of the throat, which resemble it, especially when a laboratory examination is hard to obtain. Treatment is also considered. The book is worth reading.

---

"Gray's Anatomy." Published by Lea Brothers, 706–710 Sansom street, Philadelphia.

The profession will be interested to know, that there will shortly appear a new edition of this work, with which is bound up, in the mind of nearly every physician who uses the English language, memories of many days and nights of anxious reading, and many hopes and fears as examination days drew nigh; that the old familiar pages have been further embellished by 135 new engravings; that part of the book has been rewritten, and that it now possesses the—to the mind of the student—somewhat terrible advantage of seventy-five new pages.

### MOODY'S MAGAZINE OF MEDICINE.

Another monthly medical journal has been launched in Atlanta. It is edited by Dr. R. H. Bell; Mr. J. T. Moody is the President and Business Manager, and Mr. J. J. Disosway is Vice-President and Treasurer. This magazine is unusual in several of its features. It publishes illustrations and biographies of some of its contributors, along with a picture called a "Lazy Day," which has a bearing on medicine inasmuch as it represents a striking specimen of feminine anatomy; and a picture of a railway president. It aims also at being political, and takes up the cause of Cuba; and commercial, containing paragraphs in connection with haberdashery, tobacco, etc. Poetry adds sentiment to its pages; while it contains departments intended for feminine perusal, and the insertion of short stories. In the first issue the tale is termed the "Moonshiner's Daughter," and is written by Lottie George. A couple of pages are devoted to a description of a fishing excursion and color photography. Still another link with the non-medical world is found in the "Railway Official Department," in which we have articles on the financial statement of the Atlanta and West Point and Western Railway of Alabama, on the railroad as an industrial factor, and on railway promotions.

While holding out the hand of fellowship to all medical journals tending to the benefit and elevation of our profession, and heartily commending the scientific papers in this special magazine, we, in no carping spirit, and considering only the dignity of the profession, venture to express a doubt as to the propriety of the above departures from conservative medical journalism.

---

THE AMERICAN ACADEMY OF RAILWAY SURGEONS: Report of the Second Annual Meeting Held at Chicago, Ill., September 25, 26, 27, 1895. Edited by J. Harvey Reed, M.D., Columbus, Ohio.

This book, of some two hundred pages, contains, besides the general proceedings of this meeting, the papers read there. It is well illustrated and printed, and should be valuable to those interested in the accidents incident to railway traveling.

IN SICKNESS AND IN HEALTH. A Manual of Domestic Medicine and Surgery, Hygiene, Dietetics, and Nursing. Dealing in a practical way with the problems relating to the maintenance of health, the prevention and treatment of disease, and the most effective aid in emergencies. Edited by J. West Roosevelt, M.D., late Physician in Charge of Seaton Hospital for Consumptives; Visiting Physician to Bellevue Hospital, and Attending Physician to Roosevelt Hospital, New York. Complete in one volume of over a thousand pages, illustrated with four colored plates and numerous engravings. Full analytical index. *Sold by Appleton, 205–207, Gould Building, Atlanta.* Price, $6.00, in handsome cloth binding. D. Appleton & Co., publishers, 72 Fifth avenue, New York.

A great part of this book is heartily to be commended. The article on "Anatomy," by Dr. G. W. Crary, is well written and abundantly illustrated; this is true also of the article on "Physiology," by Frederic S. Lee, Ph.D. Under the title of "Psychology," Professor Royce, of Harvard, has contributed an interesting and very readable paper dealing with the "Mind in its Various Aspects and Processes"; while W. J. Sears takes up the study of "Muscular Exercises," and, with the help of illustrations, discusses the comparative value of the various outdoor and indoor games in their relation to age and sex, country and town. This also is an excellent paper.

Among the most valuable parts of the book is that dealing with "Hygiene," by Dr. S. T. Armstrong, of New York. This is a part of medicine which is not studied nearly enough by the laity, and if they would discontinue reading and thinking about pathology and treatment and take an interest in preventive medicine, we should have fewer pale and sickly children, a diminution in contagious disease, and a general betterment in our sanitary surroundings.

It is with the latter part of the book, except the section on "Nursing" which should properly be included in a medical book for non-professional readers, that we have some fault to find. We believe in a knowledge of physiology for all, but we do not believe in endeavoring to teach the symptoms, pathology, prognosis, and treatment in surgical, medical, and obstetrical cases to any except those who are studying professionally. This does not mean that anything but good is likely to accrue from properly conducted "ambulance lectures," where first aid to the sick and wounded is taught, but when four hundred and seventy pages are given over to sections on surgery, diseases in general, and diseases of the various organs separately, and incorporated in a book intended for the general reader, it looks to us as if an attempt were being made to crown an edifice with very insufficient foundations—to teach in one book what it takes, or should take, the real student years of hard study to acquire. The inevitable result of the possession of this book by women especially, will be to increase the number of that very unpleasant race of beings who, knowing nothing properly, think they know everything medical, and are a danger both to themselves and others.

It is true that advice to call in a doctor is thickly interspersed throughout these pages, but that does not take away from the fact that the outcome of their perusal is likely to have quite the opposite effect. With this somewhat important defect, which, however, will rather increase than diminish its sale, the book is a most excellent one. The illustrations are good, and the printing large and clear.

---

A System of Surgery. By American authors. Edited by Frederic S. Dennis, M.D., Professor of the Principles and Practice of Surgery, Bellevue Hospital Medical College, New York; President of the American Surgical Association, etc., assisted by John S. Billings, M.D., LL.D., D.C.L., Deputy Surgeon-General, United States Army. Complete in four imperial octavo volumes, containing 3652 pages, with index, 1585 engravings and 45 full-page plates in black and colors. Volume IV., 970 pages, 441 engravings, and 23 plates. Price per volume, $6.00 in cloth; $7.00 in leather; $8.50 in half Morocco, gilt back and top. For sale by subscription. Full circular free to any address on application to the publishers, Lea Brothers & Co., Philadelphia.

The subscribers to this excellent system of surgery will welcome volume IV which has recently come from the press. In it Dr. Maurice H. Richardson, of Harvard, writes on the Surgery of the Stomach and Intestines, the use of the Murphy Button, Dennis Plates, etc.; McBurney, of New York, on Appendecitis; Lusk, on Symphyseotomy; W. W. Keen, of Jefferson, on the Röntgen Rays; F. S. Dennis, on Tumors; W. T. Bull, on Hernia; L. S. Pilcher, on the Surgery of the Alimentary Canal; Robert Abbe, on the Surgery of the Liver; W. M. Polk, on Diseases of the Uterus; J. T. Johnson, on the Surgery of the Ovaries and Tubes; H. C. Coe, on Minor Gynecological Surgery; Robert F. Weir, on the Surgery of the Thyroid Gland; and R. Matas, on the Surgical Peculiarities of the Negro.

These names speak for themselves. The illustrations, many of which are colored, are numerous, clear, very carefully and beautifully executed, and aid greatly in completing the value of this most excellent volume, which we have pleasure in highly recommending.

